# The Royal Orthopædic Hospital

**Published:** 1902-05-03

**Authors:** 


### Editor's Letter-Box.

### THE ROYAL ORTHOPÆDIC HOSPITAL.

Mr. H. A. Reeves writes drawing attention to certain statements in our report of the meeting at the Orthopædic Hospital which he considers do not properly represent what he said. In so writing, however, he has been led to touch upon many other topics, and in a manner which has evidently roused in his mind certain misgivings as to the law of libel, for he appends to his letter the somewhat naive request, "You will please strike out anything you think actionable in this letter, and oblige." Under the circumstances we think it best not to publish the letter.

